# Prognostic Significance of Homocysteine Levels in Patients with ST-Segment Elevation Myocardial Infarction Undergoing Primary Percutaneous Coronary Intervention: A Propensity Score Matching and Weighting Analysis

**DOI:** 10.31083/RCM25518

**Published:** 2025-02-19

**Authors:** Qianfeng Xiong, Shaoyong Chen, Junke Luo, Pengfeng Xiong, Zhenyun Nie, Lei Huang, Yao Wang, Zhen Lei, Lihui Zhang, Jing Wang

**Affiliations:** ^1^Department of Cardiology, Fengcheng People’s Hospital, The Affiliated Fengcheng Hospital of Yichun University, 331100 Fengcheng, Jiangxi, China; ^2^Department of Cardiac Intensive Care Unit, Fengcheng People’s Hospital, The Affiliated Fengcheng Hospital of Yichun University, 331100 Fengcheng, Jiangxi, China; ^3^Department of Thoracic Surgery, Fengcheng People’s Hospital, The Affiliated Fengcheng Hospital of Yichun University, 331100 Fengcheng, Jiangxi, China; ^4^Health Care Bureau, Health Commission of Shanxi Province, 030032 Taiyuan, Shanxi, China; ^5^Department of Cardiology, The Third Clinical Medical College of Shanxi Medical University, 030032 Taiyuan, Shanxi, China; ^6^Prevention & Healthcare Department, Shanxi Bethune Hospital, Shanxi Academy of Medical Sciences, Tongji Shanxi Hospital, Third Hospital of Shanxi Medical University, 030032 Taiyuan, Shanxi, China

**Keywords:** homocysteine, ST-segment elevation myocardial infarction, primary percutaneous coronary intervention, major adverse cardiac events, propensity score

## Abstract

**Background:**

Elevated homocysteine (Hcy) levels have been linked to poorer outcomes in acute coronary syndrome. This study aimed to assess the predictive value of elevated Hcy levels for major adverse cardiac events (MACE) in patients with ST-segment elevation myocardial infarction (STEMI) undergoing primary percutaneous coronary intervention (PCI).

**Methods:**

This retrospective cohort study included 183 STEMI patients who underwent primary PCI at a tertiary university hospital in southern China from January 2020 to December 2021. Laboratory values, including Hcy levels, were obtained within 24 hours of admission. Patients were categorized into elevated and normal Hcy groups using a threshold of 12 μmol/L. The study outcome was the occurrence of 6-point MACE, defined as cardiac death, nonfatal myocardial infarction, stroke, ischemia-driven revascularization (PCI or coronary artery bypass grafting), heart failure and all-cause death. Survival analyses were conducted using Kaplan-Meier and Cox proportional hazard methods. Propensity score matching (PSM) and inverse probability of treatment weighting (IPTW) approaches were employed to minimize bias.

**Results:**

The mean age of the patients was 64.8 years, with 76.0% being male. After adjusting with PSM or IPTW, covariate imbalances between the two groups were corrected. Over a median follow-up period of 25.8 months, 55 MACE events occurred, resulting in an event rate of 30.1%. Patients with elevated Hcy levels had a higher incidence of MACE in both unadjusted (hazard ratio [HR] = 2.778; 95% confidence interval [CI]: 1.591–4.850; *p <* 0.001) and adjusted analyses (PSM: HR = 2.995; 95% CI: 1.397–6.423, *p =* 0.005; IPTW: HR = 3.2; 95% CI: 1.631–6.280, *p <* 0.001). Multivariate Cox regression further confirmed that elevated Hcy levels were associated with a worse prognosis across the entire cohort (HR = 1.062, 95% CI: 1.029–1.097, *p <* 0.001), PSM cohort (HR = 1.089, 95% CI: 1.036–1.145, *p <* 0.001), and IPTW cohort (HR = 1.052, 95% CI: 1.020–1.086, *p =* 0.001).

**Conclusions:**

Elevated plasma levels of Hcy (≥12 μmol/L) are associated with worse outcomes in STEMI patients undergoing primary PCI, highlighting the potential role of Hcy as a prognostic marker in this population.

## 1. Introduction

Acute coronary syndrome (ACS), which affects millions of people annually, encompasses three types of coronary artery disease (CAD) and leads to approximately 7 million new diagnoses worldwide each year [1]. ST-segment elevation myocardial infarction (STEMI), the most critical type of ACS, constitutes about 36% of all ACS cases and results from the rupture of a vulnerable atherosclerotic plaque in a coronary artery, leading to complete occlusion and blockage of blood flow [1]. Accordingly, timely coronary catheterization and percutaneous coronary intervention (PCI) are the most effective strategies to reduce mortality in STEMI patients. However, some patients will still experience certain adverse events, such as heart failure, stroke, recurrent myocardial infarction, and even death [2]. Although traditional risk factors are still the main focus for risk stratification, emerging biomarkers like homocysteine (Hcy) hold promise for more accurately predicting residual risk and improving the efficacy of secondary prevention measures [3].

Discovered almost a century ago, Hcy is a sulfur-containing amino acid that undergoes metabolism via two main pathways: remethylation, which transform Hcy into methionine, and transsulfuration, which converts it into cysteine. Hcy levels can be significantly elevated due to deficiencies in dietary folate and B vitamins, as well as in individuals with the *MTHFR 677 TT *genotype. Conversely, B vitamin supplementation, including folic acid, B6, and B12, can lower Hcy levels. Several factors such as nutrition, age, gender, ethnicity, and genetics may also influence Hcy levels [4].

In the 1960s, pathologist Kilmer McCully made the seminal clinical observation that connected elevated plasma Hcy concentrations with vascular disease. This led him to propose the “homocysteine theory of atherosclerosis” [5], which spurred epidemiological studies revealing significant associations between even slight elevations in blood homocysteine and major vascular diseases including cardiovascular, cerebrovascular, and peripheral vascular diseases [4]; more recent studies, particularly those conducted in Chinese populations, have further corroborated these findings, demonstrating that hyperhomocysteinemia is an independent risk factor for CAD and highlighting its predictive value for obstructive CAD [6, 7].

Nonetheless, the relationship between Hcy levels and adverse outcomes in ACS remains unclear. This study, therefore, sought to evaluate the prognostic significance of elevated Hcy levels in predicting major adverse cardiac events (MACE) among STEMI patients undergoing primary PCI.

## 2. Methods

### 2.1 Study Population

This single-center retrospective cohort study involved 183 consecutive STEMI patients who underwent primary PCI at The Affiliated Fengcheng Hospital of Yichun University, a tertiary care institution in southern China, from January 2020 to December 2021. The study protocol adhered to the 1975 Declaration of Helsinki and was approved by the Ethics Committee of The Affiliated Fengcheng Hospital of Yichun University (Approval No. 202256). A threshold of 12 µmol/L was set to define hyperhomocysteinemia [8]. Based on this cutoff, patients were categorized into two groups: the normal Hcy group, with a median Hcy level of 8.7 µmol/L (range: 6.9–10.0 µmol/L), and the elevated Hcy group, with a median Hcy level of 16.6 µmol/L (range: 14.3–18.9 µmol/L). STEMI was defined by the presence of the following criteria: (1) prolonged chest pain (≥20 minutes); (2) based on the Fourth Universal Definition of Myocardial Infarction, acute STEMI on an electrocardiogram is characterized by new ST-segment elevation at the J-point in two adjacent leads. The required elevation is generally ≥1 mm, except in leads V2–V3, where the thresholds are: ≥2 mm for men aged 40 and above, ≥2.5 mm for men under 40, and ≥1.5 mm for women, regardless of age [9]; (3) elevated cardiac biomarkers above the accepted threshold [1]. Patients were excluded if they met any of the following criteria: use of folic acid supplements, presence of cancer, acute or chronic inflammatory diseases, previous history of STEMI, or prior coronary revascularization. Primary PCI, defined as percutaneous coronary intervention to the culprit artery within 12 hours of symptom onset without preceding thrombolytic therapy, was performed by an experienced team within 120 minutes of first medical contact (FMC) [10].

### 2.2 Data Collection

Two authors independently reviewed hospital medical records and the China Chest Pain Center Data Reporting Platform to collect baseline data for each patient, including demographic characteristics, laboratory findings, medical history, and angiographic and procedural details. Demographic and clinical characteristics included age, sex, body mass index (BMI), heart rate, blood pressure, Killip classification, left ventricular ejection fraction (LVEF), and laboratory results. Laboratory results primarily included blood tests conducted within 24 hours of admission, with troponin representing the highest value within 72 hours of admission, and LVEF reflecting the lowest value during hospitalization. Collected medical history data consisted of current smoking status, alcohol consumption, diabetes, hypercholesterolemia, hypertension, and chronic obstructive pulmonary disease (COPD). The diagnostic criteria for baseline characteristics were defined as follows: Hypertension was defined as having a systolic blood pressure ≥140 mmHg, diastolic blood pressure ≥90 mmHg, or the current use of antihypertensive drugs. Diabetes was identified by a fasting plasma glucose level ≥7.0 mmol/L (126 mg/dL), the use of glucose-lowering agents, or a previous diagnosis by a healthcare professional. Hypercholesterolemia was considered present if the total cholesterol level was ≥5.2 mmol/L (200 mg/dL) or if lipid-lowering therapy was being used. COPD was defined by a post-bronchodilator forced expiratory volume in one second (FEV1) to forced vital capacity (FVC) ratio of less than 0.70. Documented procedural characteristics included door-to-wire time, culprit vessel anatomy, and preoperative thrombolysis in myocardial infarction (TIMI) flow grading of the culprit lesion. Single-vessel disease was defined as having at least 50% stenosis in one major coronary artery, while multivessel disease was characterized by at least 50% stenosis in two or more major coronary arteries with a diameter of 2.5 mm or more.

### 2.3 Study Outcome

The primary endpoint was the incidence of MACE, which included cardiac death, nonfatal myocardial infarction, stroke, ischemia-driven revascularization (PCI or coronary artery bypass grafting [CABG]), heart failure and all-cause death. Stroke was defined as a sudden onset of neurological deficit caused by ischemia or hemorrhage within the brain, confirmed by clinical presentation and imaging studies. Heart failure is defined as a clinical syndrome characterized by symptoms and/or signs resulting from functional or structural cardiac abnormalities, typically confirmed by echocardiographic findings, evidence of pulmonary or systemic congestion, and/or elevated serum biomarkers such as proB-type natriuretic peptide (proBNP) [11]. Ischemia-driven revascularization was defined as revascularization procedures, such as PCI or CABG, prompted by symptoms or objective evidence of myocardial ischemia, which includes significant stenosis (≥70%) in a major coronary artery or ≥50% in the left main coronary artery as determined by coronary angiography, or a fractional flow reserve (FFR) ≤0.80 [12]. Follow-up assessments were conducted at 1, 3, and 6 months, followed by annual evaluations, with the final check-up scheduled for April 2024. These follow-ups were performed via telephone or scheduled outpatient visits to monitor disease progression and the incidence of MACE.

### 2.4 Statistical Analysis

All statistical analyses were performed using R software (version 4.3.2, R Foundation for Statistical Computing, Vienna, Austria) and SPSS software (version 22.0, IBM Corporation, Armonk, NY, USA). The Shapiro-Wilk test was employed to evaluate the normality of continuous variables. Variables following a normal distribution were summarized as mean (standard deviation) and compared between groups using an independent *t*-test. Those that did not follow a normal distribution were presented as median (Q1–Q3) and analyzed with the Mann-Whitney U test. Categorical variables were described as frequencies (percentages) and assessed using the chi-square test. Variables with less than 10% missing data in both groups were imputed using multiple imputation.

To adjust for between-group differences, propensity score-based methods were utilized, including propensity score matching (PSM) and inverse probability of treatment weighting (IPTW) [13]. For PSM, a greedy nearest-neighbor matching algorithm with a caliper width of 0.2 was applied for 1:1 matching. All covariates were initially considered for matching in PSM. To further validate our findings, we conducted sensitivity analyses using different sets of covariates, as detailed in **Supplementary Table 1**. For IPTW, the inverse propensity score was used as the weight for patients with elevated Hcy levels, and 1 minus the inverse propensity score was used as the weight for patients with normal Hcy levels. We also conducted a sensitivity analysis with E-values to evaluate the robustness of our results against potential unmeasured confounders [14]. The E-value quantifies the minimum association strength that an unmeasured confounder would require with both the exposure (elevated Hcy levels) and the outcome (MACE) to completely account for the observed relationship.

Survival analysis was conducted using Kaplan-Meier curves and Cox proportional hazards models to evaluate the association between elevated Hcy levels and MACE in STEMI patients undergoing primary PCI. Kaplan-Meier survival estimates were compared using the log-rank test. Univariate Cox regression analysis was initially conducted, and variables with a *p*-value < 0.05 were included in the multivariate Cox regression analysis to identify independent risk factors for MACE. Subgroup analysis was performed to evaluate the association between elevated Hcy levels and MACE across different subgroups, defined by baseline characteristics such as sex, age, BMI, smoking status, alcohol consumption, hypertension, diabetes, and LVEF (LVEF ≥45% or <45%). Cox proportional hazards models were used to estimate the hazard ratio (HR) and 95% confidence interval (CI) within each subgroup, and interaction terms were included to assess potential effect modification, with *p*-values reported for interaction tests. A two-tailed *p*-value < 0.05 was considered statistically significant.

## 3. Results

**Fig. 1.**
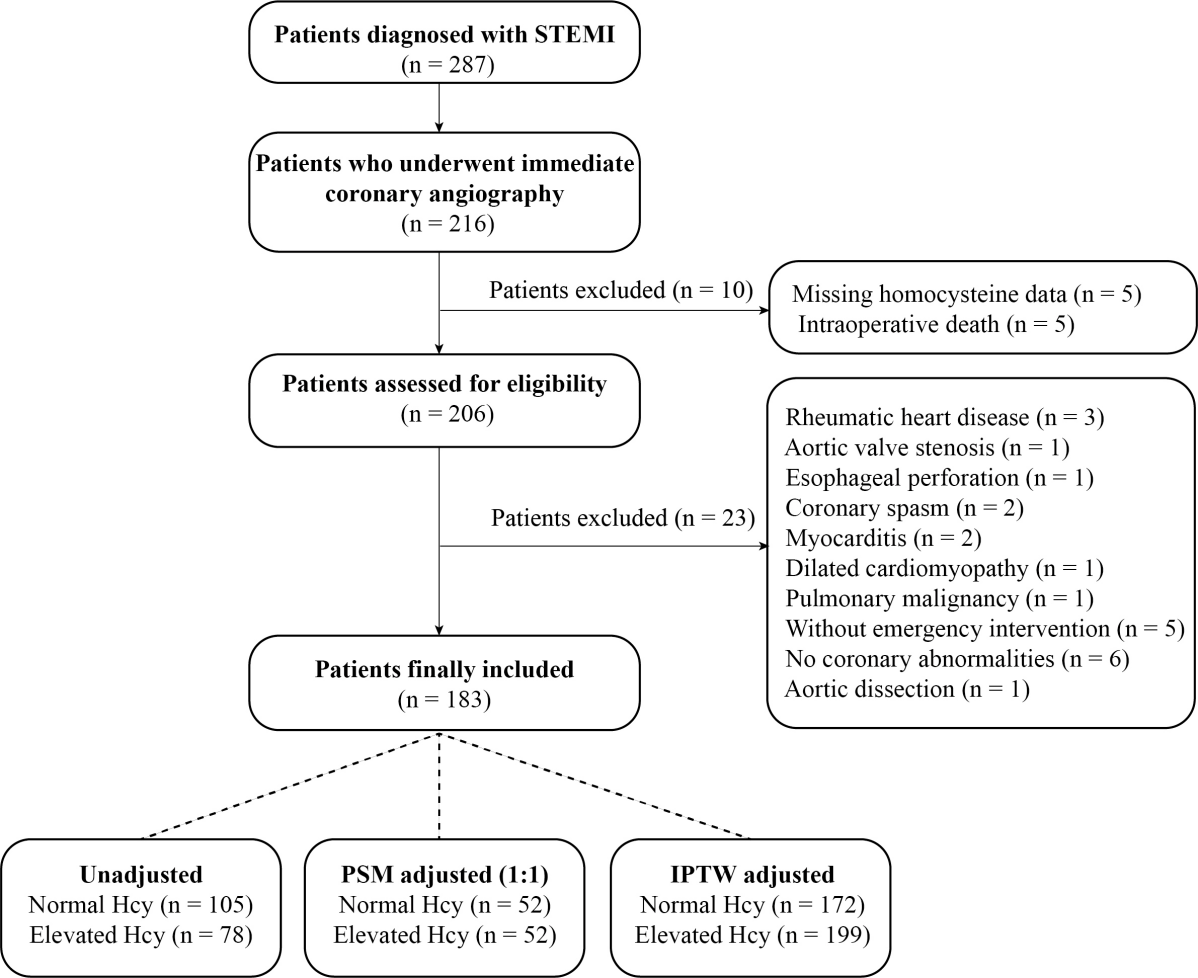
**Study flow diagram**. Abbreviations: STEMI, ST-segment elevation myocardial infarction; Hcy, homocysteine; PSM, propensity score matching; IPTW, inverse probability of treatment weighting.

A total of 183 STEMI patients who underwent primary PCI were included in the final analysis of this study (Fig. 1). Among these patients, 76.0% were male, and the mean age was 64.8 ± 12.4 years. 105 patients were in the normal Hcy level group and the remaining 78 patients were in the elevated Hcy group. The overall Hcy level was 11.0 µmol/L (8.4, 15.2), with a median level of 8.7 µmol/L (6.9, 10.0) in the normal Hcy group and 16.6 µmol/L (14.3, 18.9) in the elevated Hcy group, respectively. The two groups had similar baseline clinical characteristics, medical histories and angiographic findings (Table 1). However, there were significant differences in several clinical characteristics, such as laboratory findings and medical history, between the two groups. Patients in the elevated Hcy group had higher serum creatinine levels and a greater prevalence of hypertension and smoking, whereas those in the normal Hcy group exhibited higher levels of total cholesterol and low-density lipoprotein cholesterol.

**Table 1.** **Baseline clinical characteristics according to homocysteine level**.

			Overall (n = 183)	Normal Hcy (n = 105)	Elevated Hcy (n = 78)	*p*-value
Demographics						
	Age, year		64.8 (12.4)	63.6 (12.8)	66.4 (11.7)	0.128
	Male, n (%)		139 (76.0%)	75 (71.4%)	64 (82.1%)	0.688
	BMI, kg·m^-2^		23.8 (3.2)	23.8 (3.2)	23.6 (3.0)	0.634
	HR, bpm		73 (63, 86)	74.0 (63, 86)	73.0 (65, 87)	0.766
	SBP, mmHg		131 (113, 147)	131 (113, 150)	131 (110, 145)	0.459
	DBP, mmHg		81 (70, 94)	81 (73, 94)	80 (68, 91)	0.176
	Troponin, ng/mL		28.8 (10.7, 50.0)	28.4 (13.0, 50.0)	31.6 (8.7, 50.0)	0.402
	NT-proBNP, pg/mL		1314.0 (323.0, 2852.1)	1085.1 (306.2, 3208.0)	1368.2 (349.4, 2642.8)	0.548
	TC, mmol/L		4.6 (1.1)	4.8 (1.1)	4.4 (1.0)	0.022
	TG, mmol/L		1.1 (0.7, 1.5)	1.1 (0.8, 1.6)	1.0 (0.7, 1.4)	0.056
	HDL-C, mmol/L		1.1 (1.0, 1.4)	1.2 (1.0, 1.4)	1.1 (1.0, 1.3)	0.280
	LDL-C, mmol/L		2.9 (0.8)	3.0 (0.8)	2.8 (0.9)	0.046
	WBC, 10^9^/L		10.4 (8.4, 12.9)	11.3 (8.7, 13.3)	10.0 (7.9, 12.1)	0.062
	RBC, 10^12^/L		4.2 (0.6)	4.2 (0.6)	4.1 (0.6)	0.216
	PLT, 10^9^/L		203.0 (173.5, 249.0)	206.0 (182.0, 250.0)	197.0 (170.3, 243.3)	0.223
	HB, g/L		132.0 (116.0, 144.0)	132.0 (119.0, 145.0)	130.0 (113.3, 142.8)	0.259
	HCT, %		38.3 (32.5, 41.9)	38.8 (33.3, 42.1)	36.7 (31.2, 40.9)	0.194
	D-dimer, mg/L		0.4 (0.3, 0.6)	0.4 (0.2, 0.6)	0.4 (0.3, 0.7)	0.378
	Serum creatinine, µmol/L		70.0 (60.0, 87.0)	66.1 (57.2, 76.9)	76.7 (65.9, 102.0)	<0.001
	Na^+^, mmol/L		139.0 (136.8, 141.0)	138.6 (136.8, 140.3)	139.7 (137.0, 141.5)	0.122
	K^+^, mmol/L		3.9 (3.7, 4.2)	3.9 (3.7, 4.2)	4.0 (3.7, 4.3)	0.311
	Uric acid, µmol/L		363.0 (125.4)	350.5 (114.5)	379.8 (137.7)	0.119
	LVEF ≥45%, n (%)		170 (92.9)	96 (91.4)	74 (94.9)	0.545
	Killip classification ≥2, n (%)		39 (21.3)	24 (22.9)	15 (19.2)	0.682
Medical history						
	Smoking, n (%)		101 (55.2)	50 (47.6)	51 (65.4)	0.025
	Alcohol, n (%)		32 (17.5)	17 (16.2)	15 (19.2)	0.735
	Hypertension, n (%)		108 (59.0)	55 (52.4)	53 (68.0)	0.049
	Diabetes, n (%)		47 (25.7)	27 (25.7)	20 (25.6)	1
	Hyperlipidemia, n (%)		28 (15.3)	18 (17.1)	10 (12.8)	0.551
	COPD, n (%)		20 (10.9)	8 (7.6)	12 (15.4)	0.154
Angiographic characteristics						
	D2W, mins		80 (62, 105)	83 (62, 106)	79 (63, 100)	0.715
	Culprit lesion					0.640
		RCA, n (%)	72 (39.3)	40 (38.1)	32 (41.0)	
		LAD, n (%)	91 (49.7)	55 (52.4)	36 (46.2)	
		LCX, n (%)	20 (10.9)	10 (9.5)	10 (12.8)	
	Multivessel disease, n (%)		116 (63.4)	70 (66.7)	46 (59.0)	0.361
	Preprocedural TIMI flow ≥2, n (%)		51 (27.9)	27 (25.7)	24 (30.8)	0.557
	Number of stents ≥2, n (%)		82 (44.8)	51 (48.6)	31 (39.7)	0.300

**Notes: **Data are presented as mean (standard deviation), median (inter-quartile range), or n (%). **Abbreviations: **BMI, body mass index; HR, heart rate; SBP, systolic blood pressure; DBP, diastolic blood pressure; NT-proBNP, N-terminal proB-type natriuretic peptide; TC, total cholesterol; TG, triglycerides; HDL-C, high-density lipoprotein cholesterol; LDL-C, low-density lipoprotein cholesterol; WBC, white blood cell; RBC, red blood cell; PLT, platelet count; HB, hemoglobin; HCT, hematocrit; LVEF, left ventricular ejection fraction; COPD, chronic obstructive pulmonary disease; D2W, door-to-wire time; RCA, right coronary artery; LAD, left anterior descending; LCX, left circumflex artery; TIMI, thrombolysis in myocardial infarction; Hcy, homocysteine.

**Fig. 2.**
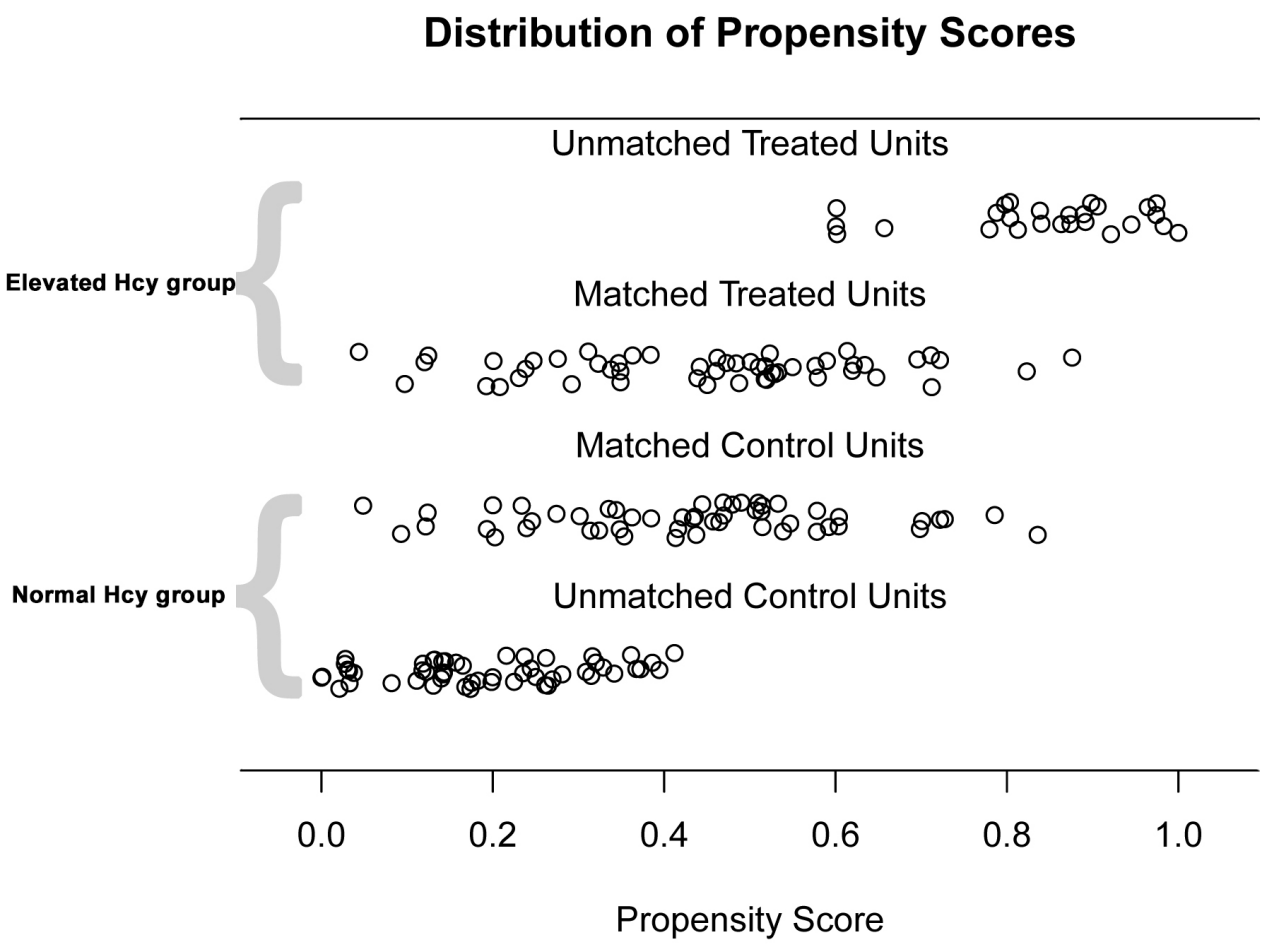
**Jitter plot of propensity score matching (PSM)**. This figure illustrates the distribution of propensity scores before and after matching between the elevated Hcy group (treated units) and the normal Hcy group (control units). “Unmatched Treated Units” and “Unmatched Control Units” represent the propensity scores of patients before matching, while “Matched Treated Units” and “Matched Control Units” indicate the scores after PSM. Hcy, homocysteine.

After adjustment with the PSM or IPTW method, baseline covariates were balanced between the groups (Table 2). The propensity score jitter plot demonstrates that the distribution of propensity scores is well balanced following PSM adjustment (Fig. 2).

**Table 2.** **Demographic, medical history, and angiographic characteristics in PSM and IPTW adjusted analysis**.

			PSM adjusted		*p*-value	IPTW adjusted		*p*-value
			Normal Hcy (n = 52)	Elevated Hcy (n = 52)		Normal Hcy (n = 172)	Elevated Hcy (n = 199)	
Demographics								
	Age, year		64.6 (13.0)	65.7 (12.2)	0.659	64.9 (12.7)	68.6 (15.1)	0.376
	Male, n (%)		42 (80.8)	40 (76.9)	0.810	125.1 (72.8)	134.9 (68.0)	0.663
	BMI, kg·m^-2^		23.4 (3.1)	23.7 (2.9)	0.542	23.5 (3.2)	23.4 (2.9)	0.828
	HR, bpm		77 (63, 85)	73 (65, 86)	0.782	74 (62, 88)	75 (66, 88)	0.717
	SBP, mmHg		130 (118, 149)	134 (112, 14)	0.656	130 (113, 145)	131 (106, 152)	0.868
	DBP, mmHg		80 (72, 94)	84 (74, 91)	0.940	80 (72, 92)	82 (69, 94)	0.798
	Troponin, ng/mL		28.0 (11.6, 50.0)	32.4 (9.9, 50.0)	0.675	26.7 (9.3, 50.0)	44.7 (11.1, 50.0)	0.293
	NT-proBNP, pg/mL		1010.2 (314.6, 2285.8)	1386.3 (315.4, 2761.3)	0.656	1071.1 (254.5, 3420.8)	1599.7 (459.1, 4699.5)	0.250
	TC, mmol/L		4.6 (1.0)	4.52 (1.1)	0.879	4.6 (1.1)	4.7 (1.1)	0.675
	TG, mmol/L		1.0 (0.7, 1.5)	1.0 (0.7, 1.5)	0.861	1.0 (0.7, 1.5)	1.0 (0.7, 1.4)	0.829
	HDL-C, mmol/L		1.1 (1.0, 1.4)	1.2 (1.1, 1.4)	0.580	1.1 (1.0, 1.4)	1.1 (0.9, 1.3)	0.369
	LDL-C, mmol/L		2.9 (0.8)	2.9 (0.9)	0.984	2.9 (0.9)	3.0 (1.0)	0.748
	WBC, 10^9^/L		10.5 (8.4, 13.2)	10.2 (8.3, 12.4)	0.772	11.2 (8.6, 13.1)	10.6 (9.0, 15.0)	0.649
	RBC, 10^12^/L		4.2 (0.7)	4.1 (0.5)	0.564	4.1 (0.7)	4.1 (0.6)	0.951
	PLT, 10^9^/L		207.5 (176.0, 240.0)	200.5 (173.8, 241.8)	0.974	205.0 (173.7, 249.5)	194.5 (176.3, 241.6)	0.615
	HB, g/L		133.0 (118.3, 145.0)	130.5 (115.5, 142.3)	0.491	132.0 (117.0, 144.4)	126.9 (112.0, 140.0)	0.283
	HCT, %		38.6 (32.0, 42.0)	36.7 (32.4, 39.6)	0.246	38.5 (30.0, 41.8)	37.0 (32.8, 39.3)	0.475
	D-dimer, mg/L		0.4 (0.3, 0.5)	0.4 (0.3, 0.6)	0.644	0.4 (0.3, 0.6)	0.4 (0.3, 0.9)	0.286
	Serum creatinine, µmol/L		72.2 (62.9, 86.1)	70.5 (60.7, 86.9)	0.984	69.9 (59.0, 83.0)	70.0 (65.0, 80.9)	0.421
	Na^+^, mmol/L		138.2 (136.7, 140.4)	139.1 (137.2, 141.1)	0.235	138.5 (136.7, 140.3)	138.9 (137.2, 141.2)	0.609
	K^+^, mmol/L		3.9 (3.7, 4.3)	4.0 (3.7, 4.2)	0.718	3.9 (3.6, 4.2)	3.9 (3.8, 4.1)	0.873
	Uric acid, µmol/L		364.7 (110.1)	364.6 (137.4)	0.997	355.5 (279.8, 437.8)	382.63 (286.9, 423.7)	0.393
	LVEF ≥45%, n (%)		49 (94.2)	48 (92.3)	1	158.54 (92.3)	162.9 (82.0)	0.229
	Killip classification ≥2, n (%)		10 (19.2)	11 (21.2)	1	36.84 (21.4)	48.72 (24.5)	0.713
Medical history								
	Smoking, n (%)		33 (63.5)	32 (61.5)	1	91.1 (53.0)	107.3 (54.1)	0.923
	Alcohol, n (%)		11 (21.2)	9 (17.3)	0.804	28.3 (16.5)	31.7 (16.0)	0.939
	Hypertension, n (%)		30 (57.7)	32 (61.5)	0.842	93.0 (54.1)	101.6 (51.2)	0.778
	Diabetes, n (%)		12 (23.1)	10 (19.2)	0.81	38.0 (22.1)	37.9 (19.1)	0.651
	Hyperlipidemia, n (%)		8 (15.4)	7 (13.5)	1	25.0 (14.6)	27.3 (13.8)	0.898
	COPD, n (%)		6 (11.5)	5 (9.6)	1	13.7 (8.0)	18.0 (9.1)	0.790
Angiographic characteristics								
	D2W, mins		84 (62, 102)	78 (64, 98)	0.770	78.0 (57.6, 98.0)	80.0 (66.0, 97.1)	0.677
	Culprit lesion				0.815			0.947
		RCA, n (%)	20 (38.5)	20 (38.5)		63.3 (36.8)	71.0 (35.8)	
		LAD, n (%)	25 (48.1)	27 (51.9)		91.4 (53.2)	103.8 (52.3)	
		LCX, n (%)	7 (13.5)	5 (9.6)		17.2 (10.0)	23.7 (12.0)	
	Multivessel disease, n (%)		34 (65.4)	31 (59.6)	0.685	115.7 (67.4)	141.94 (71.5)	0.607
	Preprocedural TIMI flow ≥2, n (%)		17 (32.7)	17 (32.7)	1	47.1 (27.4)	64.44 (32.5)	0.634
	Number of stents ≥2, n (%)		28 (53.9)	23 (44.2)	0.433	79.8 (46.5)	105.47 (53.1)	0.511

**Notes: **Data are presented as mean (standard deviation), median (inter-quartile range), or n (%). **Abbreviations: **PSM, propensity score matching; IPTW, inverse probability of treatment weighting; BMI, body mass index; HR, heart rate; SBP, systolic blood pressure; DBP, diastolic blood pressure; NT-proBNP, N-terminal proB-type natriuretic peptide; TC, total cholesterol; TG, triglycerides; HDL-C, high-density lipoprotein cholesterol; LDL-C, low-density lipoprotein cholesterol; WBC, white blood cell; RBC, red blood cell; PLT, platelet count; HB, hemoglobin; HCT, hematocrit; LVEF, left ventricular ejection fraction; COPD, chronic obstructive pulmonary disease; D2W, door-to-wire time; RCA, right coronary artery; LAD, left anterior descending; LCX, left circumflex artery; TIMI, thrombolysis in myocardial infarction; Hcy, homocysteine.

### 3.1 Follow-up Outcomes

**Fig. 3.**
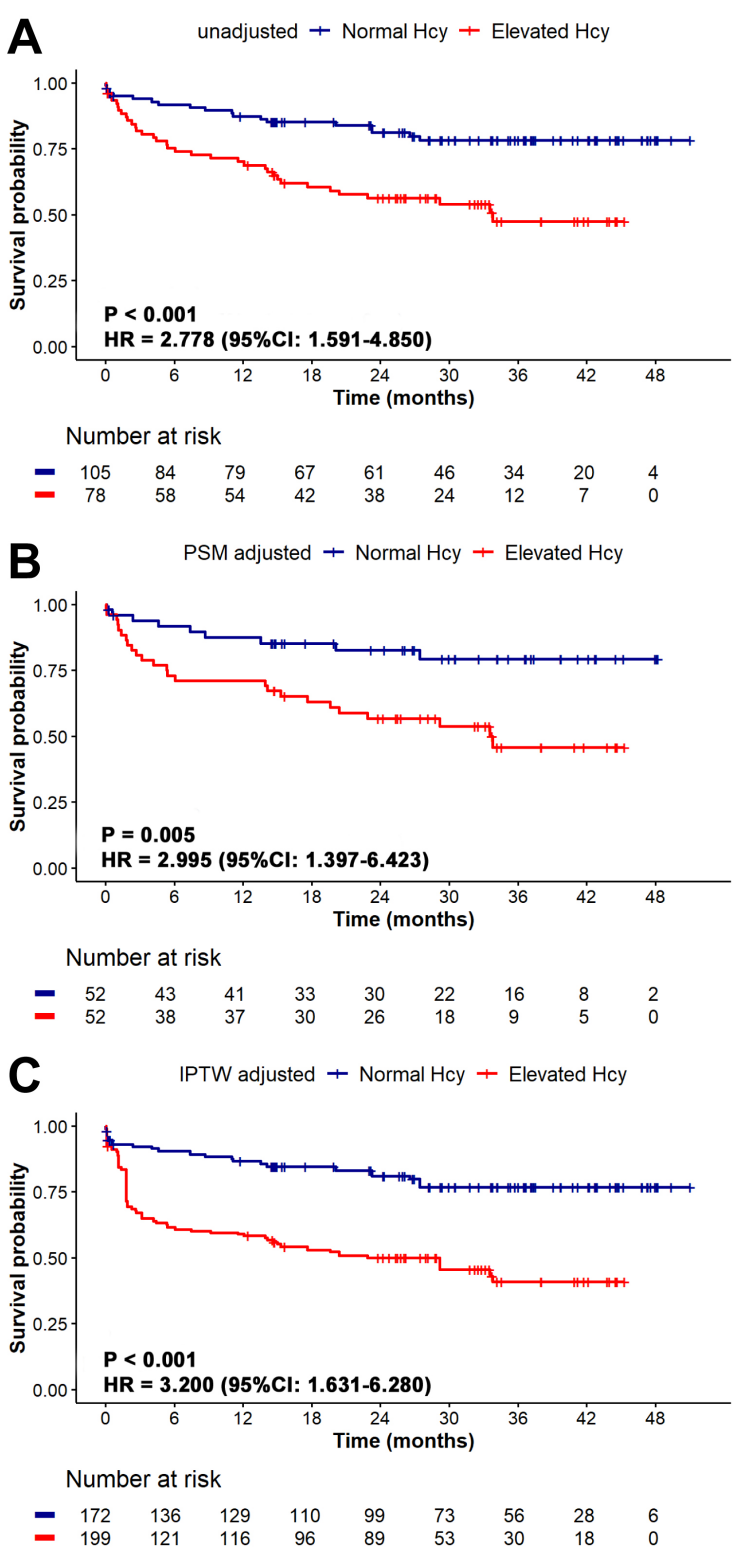
**Kaplan-Meier survival analysis for MACE according to Hcy levels: (A) unadjusted analysis, (B) PSM adjusted, (C) IPTW adjusted**. The survival analysis is performed with the use of the Cox proportional-hazard model. The Kaplan-Meier survival analysis shows that the elevated Hcy group has a lower survival probability compared to the normal Hcy group, with unadjusted, PSM-adjusted, and IPTW-adjusted analyses all confirming an increased risk of MACE associated with higher Hcy levels and demonstrating the robustness of this association after adjusting for confounding factors. Hcy, homocysteine; HR, hazard ratio; CI, confidence interval; PSM, propensity score matching; IPTW, inverse probability of treatment weighting; MACE, major adverse cardiac events.

Over a median follow-up period of 25.8 (10.1, 35.9) months, 55 patients (30.1%) experienced MACE, with 19 cases in the normal Hcy group and 36 in the elevated Hcy group. The incidence rates of different events are detailed in **Supplementary Table 2**. The Kaplan-Meier survival plot (Fig. 3) demonstrated a significantly higher incidence of MACE in the elevated Hcy group according to the Cox proportional hazard model (HR = 2.778, 95% CI: 1.591–4.850, *p* < 0.001). This increased incidence remained significant in the elevated Hcy group even after adjustments using PSM (HR = 2.995, 95% CI: 1.397–6.423, *p* = 0.005) and IPTW (HR = 3.2, 95% CI: 1.631–6.280, *p* < 0.001).

### 3.2 Sensitivity Analysis

The E-values for the unadjusted, PSM, and IPTW analyses were 5.000, 5.439, and 5.853, respectively, suggesting that an unmeasured confounder would require a strong association with both Hcy levels and MACE to fully explain away these findings (**Supplementary Table 3**). These findings support the robustness of the observed association between elevated Hcy levels and an increased risk of MACE, even in the presence of potential unmeasured confounders.

### 3.3 Subgroup Analysis

Subgroup analysis revealed varying associations between elevated homocysteine levels and MACE across different subgroups (**Supplementary Fig. 1**). The P-interaction values indicated no significant interaction effects among subgroups (all *p* > 0.05), demonstrating that our main findings were consistent across subgroups defined by age (<60 or ≥60), sex, BMI (<25 or ≥25), smoking status, alcohol consumption, hypertension, and diabetes.

### 3.4 Cox Multivariate Analysis

In the multivariate Cox proportional hazards analysis, age (HR = 1.052, 95% CI: 1.023–1.081, *p* < 0.001), Hcy (HR = 1.062, 95% CI: 1.029–1.097, *p* < 0.001), and LVEF ≥45% (HR = 0.251, 95% CI: 0.116–0.543, *p* < 0.001) were identified as independent predictors of MACE (Table 3). Even after adjustments with PSM or IPTW, Hcy remained a significant risk factor in STEMI patients undergoing primary PCI (PSM adjusted: HR = 1.089, 95% CI: 1.036–1.145, *p* < 0.001; IPTW adjusted: HR = 1.052, 95% CI: 1.020–1.086, *p* = 0.001). Furthermore, both age (PSM adjusted: HR = 1.045, 95% CI: 1.012–1.079, *p* = 0.008; IPTW adjusted: HR = 1.051, 95% CI: 1.019–1.084, *p* = 0.002), and low-density lipoprotein cholesterol (LDL-C) (PSM adjusted: HR = 1.866, 95% CI: 1.162–2.996, *p* = 0.010; IPTW adjusted: HR = 2.765, 95% CI: 1.692–4.520, *p* < 0.001) were also independently associated with a worse prognosis (Table 4).

**Table 3.** **Univariate and multivariate Cox regression analysis for predicting MACE in total cohort**.

		Univariate		Multivariate	
		HR (95% CI)	*p*-value	HR (95% CI)	*p*-value
Age		1.072 (1.044–1.102)	<0.001	1.052 (1.023–1.081)	<0.001
Male		0.699 (0.391–1.251)	0.228		
BMI		0.978 (0.896–1.066)	0.609		
Heart rate		1.009 (0.996–1.022)	0.196		
SBP		1.008 (0.999–1.016)	0.098		
DBP		0.998 (0.984–1.012)	0.771		
Troponin		1.004 (0.999–1.009)	0.149		
NT-proBNP		1.001 (0.999–1.001)	0.241		
TC		1.008 (0.785–1.294)	0.952		
TG		0.781 (0.519–1.177)	0.238		
HDL-C		0.946 (0.474–1.888)	0.874		
LDL-C		1.267 (0.907–1.77)	0.166		
WBC		0.983 (0.922–1.048)	0.605		
RBC		0.474 (0.299–0.751)	0.001	1.32 (0.549–3.171)	0.535
PLT		1.003 (1–1.006)	0.068		
HB		0.974 (0.96–0.988)	<0.001	0.984 (0.954–1.014)	0.299
HCT		0.982 (0.968–0.997)	0.019	0.99 (0.972–1.008)	0.260
D-dimer		1.72 (1.227–2.413)	0.002	1.444 (0.881–2.367)	0.145
Serum creatinine		1.005 (1.001–1.009)	0.013	0.997 (0.991–1.003)	0.270
Na^+^		0.997 (0.984–1.01)	0.615		
K^+^		0.978 (0.878–1.089)	0.682		
Uric acid		1.001 (0.999–1.003)	0.357		
Hcy		1.035 (1.011–1.059)	0.004	1.062 (1.029–1.097)	<0.001
LVEF ≥45%		0.187 (0.094–0.375)	<0.001	0.251 (0.116–0.543)	<0.001
Killip classification ≥2		1.166 (0.614–2.211)	0.639		
Smoking		0.829 (0.488–1.407)	0.487		
Alcohol		1.029 (0.518–2.042)	0.935		
Hypertension		1.297 (0.744–2.262)	0.359		
Diabetes		1.106 (0.611–2.003)	0.739		
Hyperlipidemia		1.228 (0.619–2.438)	0.556		
COPD		1.711 (0.836–3.504)	0.142		
D2W		1.002 (0.997–1.008)	0.402		
Culprit lesion					
	RCA	1 (reference)			
	LAD	0.932 (0.528–1.645)	0.808		
	LCX	1.262 (0.539–2.955)	0.592		
Multivessel disease		1.047 (0.604–1.814)	0.870		
Preprocedural TIMI flow ≥2		0.736 (0.395–1.371)	0.334		
Number of stents ≥2		0.966 (0.567–1.647)	0.900		

**Notes:** Variables with a *p*-value < 0.05 in univariate analysis were included in the multivariate analysis. **Abbreviations: **BMI, body mass index; SBP, systolic blood pressure; DBP, diastolic blood pressure; NT-proBNP, N-terminal proB-type natriuretic peptide; TC, total cholesterol; TG, triglycerides; HDL-C, high-density lipoprotein cholesterol; LDL-C, low-density lipoprotein cholesterol; WBC, white blood cell; RBC, red blood cell; PLT, platelet count; HB, hemoglobin; HCT, hematocrit; LVEF, left ventricular ejection fraction; COPD, chronic obstructive pulmonary disease; D2W, door-to-wire time; RCA, right coronary artery; LAD, left anterior descending; LCX, left circumflex artery; TIMI, thrombolysis in myocardial infarction; Hcy, homocysteine; HR, hazard ratio; CI, confidence interval; MACE, major adverse cardiac events.

**Table 4.** **Univariate and multivariate Cox regression analysis for predicting MACE in PSM or IPTW adjusted cohort**.

		PSM adjusted				IPTW adjusted			
		Univariate		Multivariate		Univariate		Multivariate	
		HR (95% CI)	*p*-value	HR (95% CI)	*p*-value	HR (95% CI)	*p*-value	HR (95% CI)	*p*-value
Age		1.062 (1.029–1.097)	<0.001	1.045 (1.012–1.079)	0.008	1.065 (1.041–1.089)	<0.001	1.051 (1.019–1.084)	0.002
Male		0.727 (0.329–1.607)	0.431			0.432 (0.2–0.934)	0.033	1.383 (0.708–2.702)	0.343
BMI		0.954 (0.855–1.064)	0.395			0.952 (0.861–1.053)	0.341		
Heart rate		1.022 (1.001–1.043)	0.037	0.998 (0.97–1.027)	0.895	1.016 (0.998–1.033)	0.075		
SBP		1.010 (0.998–1.021)	0.090			1.008 (0.994–1.022)	0.271		
DBP		1.002 (0.984–1.021)	0.812			1 (0.982–1.018)	0.959		
Troponin		1.004 (0.998–1.009)	0.186			1.003 (1.001–1.007)	0.062		
NT-proBNP		1.001 (0.999–1.001)	0.649			1.001 (0.999–1.001)	0.844		
TC		1.166 (0.827–1.643)	0.381			1.27 (0.939–1.718)	0.121		
TG		0.653 (0.333–1.278)	0.214			0.777 (0.49–1.232)	0.284		
HDL-C		0.914 (0.395–2.117)	0.834			0.482 (0.177–1.313)	0.154		
LDL-C		1.663 (1.077–2.567)	0.022	1.866 (1.162–2.996)	0.010	1.729 (1.083–2.761)	0.022	2.765 (1.692–4.520)	<0.001
WBC		0.989 (0.897–1.09)	0.825			1.048 (0.99–1.11)	0.107		
RBC		0.557 (0.303–1.025)	0.060			0.586 (0.363–0.947)	0.029	1.192 (0.396–3.588)	0.754
PLT		1.003 (0.998–1.008)	0.250			1.002 (0.998–1.006)	0.263		
HB		0.982 (0.963–1.001)	0.061			0.98 (0.962–0.999)	0.035	0.968 (0.927–1.011)	0.145
HCT		0.982 (0.964–0.999)	0.048	0.988 (0.968–1.008)	0.235	0.985 (0.97–1)	0.054		
D-dimer		4.856 (2.655–8.881)	<0.001	5.306 (2.354–11.955)	<0.001	2.067 (1.36–3.142)	0.001	1.566 (0.991–2.473)	0.054
Serum creatinine		1.005 (0.998–1.011)	0.139			1.001 (0.995–1.007)	0.739		
Na^+^		1.036 (0.935–1.148)	0.499			0.998 (0.995–1.001)	0.177		
K^+^		1.361 (0.604–3.065)	0.457			0.983 (0.955–1.011)	0.228		
Uric acid		1.001 (0.998–1.004)	0.412			1 (0.997–1.003)	0.828		
Hcy		1.072 (1.027–1.118)	0.001	1.089 (1.036–1.145)	<0.001	1.047 (1.016–1.079)	0.003	1.052 (1.020–1.086)	0.001
LVEF ≥45%		0.137 (0.055–0.344)	<0.001	0.339 (0.075–1.529)	0.159	0.138 (0.07–0.274)	<0.001	0.259 (0.111–0.605)	0.002
Killip Classification ≥2		1.495 (0.677–3.304)	0.320			1.276 (0.503–3.234)	0.608		
Smoking		0.639 (0.325–1.258)	0.195			0.562 (0.275–1.149)	0.114		
Alcohol		1.709 (0.796–3.667)	0.169			1.433 (0.614–3.344)	0.406		
Hypertension		1.391 (0.678–2.855)	0.368			0.711 (0.339–1.49)	0.366		
Diabetes		1.290 (0.584–2.851)	0.529			0.84 (0.406–1.736)	0.638		
Hyperlipidemia		1.357 (0.562–3.279)	0.498			0.808 (0.349–1.875)	0.620		
COPD		1.988 (0.82–4.819)	0.128			1.297 (0.599–2.807)	0.509		
D2W, mins		1.004 (0.998–1.011)	0.165			1.004 (0.999–1.008)	0.135		
Culprit lesion									
	RCA	1 (reference)				1 (reference)			
	LAD	1.177 (0.571–2.425)	0.659			1.084 (0.479–2.454)	0.846		
	LCX	0.722 (0.203–2.562)	0.614			1.274 (0.387–4.193)	0.691		
Multivessel disease		1.280 (0.624–2.628)	0.500			1.676 (0.872–3.221)	0.121		
Preprocedural TIMI flow ≥2		0.612 (0.277–1.353)	0.225			0.971 (0.362–2.603)	0.954		
Number of stents ≥2		1.056 (0.539–2.069)	0.874			1.403 (0.68–2.892)	0.359		

**Notes:** Variables with a *p*-value < 0.05 in univariate analysis were included in the multivariate analysis. **Abbreviations: **MACE, major adverse cardiac events; PSM, propensity score matching; IPTW, inverse probability of treatment weighting; BMI, body mass index; SBP, systolic blood pressure; DBP, diastolic blood pressure; NT-proBNP, N-terminal proB-type natriuretic peptide; TC, total cholesterol; TG, triglycerides; HDL-C, high-density lipoprotein cholesterol; LDL-C, low-density lipoprotein cholesterol; WBC, white blood cell; RBC, red blood cell; PLT, platelet count; HB, hemoglobin; HCT, hematocrit; LVEF, left ventricular ejection fraction; COPD, chronic obstructive pulmonary disease; D2W, door-to-wire time; RCA, right coronary artery; LAD, left anterior descending; LCX, left circumflex artery; TIMI, thrombolysis in myocardial infarction; Hcy, homocysteine; HR, hazard ratio; CI, confidence interval.

## 4. Discussion

In this cohort of 183 STEMI patients undergoing primary PCI, we observed that those with hyperhomocysteinemia (≥12 µmol/L) experienced a higher rate of long-term MACE. After two statistical adjustment approaches for differences between two groups, the association between elevated Hcy and MACE remained evident.

Hcy, an intermediate metabolite of methionine, contributes to endothelial dysfunction and inflammation, smooth muscle cell proliferation, platelet activation, and increased risk of thrombosis [5, 15]. With the advent of high-performance liquid chromatography methods for the rapid measurement of Hcy concentrations, epidemiological studies have uncovered significant links between modest increases in blood Hcy levels and cardiovascular, cerebrovascular, and peripheral vascular diseases [16, 17]. Several *in vitro* and *in vivo* studies using pathogenic concentrations of Hcy and hyperhomocysteinemia animal models have demonstrated that Hcy induces harmful effects on both vascular resident cells and circulating leukocytes [18]. As an amino acid containing a reactive sulfhydryl group, Hcy is thought to contribute to vascular inflammation and injury by promoting oxidative stress through the accumulation of reactive oxygen species [19]. This oxidative stress can lead to endothelial dysfunction, smooth muscle cell proliferation, and vascular calcification, thereby increasing the risk of cardiac and vascular diseases [18]. Supporting this hypothesis, hyperhomocysteinemia has consistently been linked to a heightened risk of various cardiovascular conditions, including atherosclerosis, coronary heart disease, heart failure, and atrial fibrillation. Moreover, it is strongly correlated with a higher incidence of stroke and greater overall mortality [20].

Despite the strong associations mentioned above, early large-scale trials of Hcy lowering with vitamins therapy, such as the Vitamin Intervention for Stroke Prevention (VISP) [21] and the Heart Outcomes Prevention Evaluation 2 (HOPE-2) trial [22], did not demonstrate a reduction in stroke incidence. However, in 2015, the China Stroke Primary Prevention Trial (CSPPT), which included over 20,000 participants and had a five-year follow-up period, demonstrated that folic acid supplementation significantly reduced the incidence of stroke [23]. The underlying causes of these contradictory findings remain uncertain and may be linked to variations in ethnicity, genetics factors, or dietary habits. Notably, the traditional Chinese diet typically involves lower meat consumption, higher vegetable intake, and lacks folic acid fortification [24]. A meta-analysis has demonstrated a weaker association between Hcy levels and coronary heart disease compared to stroke, potentially due to differences in the pathogenesis of cardiovascular and cerebrovascular diseases [25].

Another interesting point of view is that prospective studies exhibited significantly weaker associations between homocysteine levels and the risks of ischemic heart disease compared to retrospective studies [25]. Both retrospective and prospective cohort data were included in this meta-analysis, and the target populations, follow-up times, and Hcy cut-off values varied. This discrepancy may arise from biases inherent in retrospective studies, including difficulties in selecting appropriate controls and the impact of changes in treatment and other factors following disease onset. Several studies have concluded that high Hcy levels adversely affect the prognosis of patients with ACS, increasing the risk of MACE [26, 27, 28, 29, 30, 31] and all-cause mortality [31, 32, 33], with a targeted meta-analysis [34] indicating that the risk can more than double. Conversely, other studies have concluded that homocysteine acts as a bystander rather than a causative factor and does not independently predict cardiovascular mortality in STEMI patients [35, 36]. Therefore, the heterogeneity among studies complicates the interpretation of the true effect of Hcy. Differences in race, geographic region, socioeconomic status, and dietary habits may contribute to the observed discrepancies. However, recent advancements in primary PCI, new-generation drug-eluting stents, and novel P2Y12 inhibitors are more likely to have diminished the prognostic significance of Hcy in cardiovascular outcomes. To date, it remains unknown whether Hcy level has a prognostic impact on STEMI patients undergoing primary PCI. In this study, Kaplan-Meier survival analysis demonstrated a higher incidence of MACE in the elevated Hcy group compared to the normal Hcy group, and these results remain robust after adjusting for confounders using PSM and IPTW. Subgroup analysis further confirmed the consistency of this association across various patient subgroups. The calculated E-value supported the robustness of the observed association between elevated Hcy levels and increased MACE risk, indicating that the findings are unlikely to be explained by unmeasured confounding alone. Cox regression analysis identified several factors significantly associated with prognosis, including Hcy, age, and LDL-C, indicating that these factors should be considered critical in assessing patient risk profiles.

This study has several strengths and limitations. One of the main strengths is the use of propensity score adjustments to account for various potential confounding factors, such as medical history and laboratory results. By employing two propensity score-based adjustment methods, we achieved a balanced distribution of baseline covariates between the elevated and the normal Hcy group, allowing for a more comprehensive evaluation of the results. To our knowledge, this is the first study to establish an association between Hcy levels and the risk of MACE in STEMI patients undergoing primary PCI in Southern China, a region without folic acid fortification. However, several limitations should be considered. The retrospective observational design, single-center setting, and relatively small sample size may affect the generalizability of our findings. Additionally, the absence of Hcy level monitoring during the follow-up period may have influenced the results. Although PSM and IPTW were employed to minimize selection bias, potential limitations inherent to a non-randomized study design may still be present.

## 5. Conclusions

In conclusion, elevated Hcy levels were a significant predictor of MACE in STEMI patients undergoing primary PCI. While these findings are encouraging, the current data highlight the need for future well-designed randomized controlled trials to investigate the long-term benefits of regulating Hcy levels in patients with coronary artery disease. The prevailing view is that lowering Hcy levels with folic acid and vitamin therapies does not change the prognosis for coronary artery disease [37, 38]. Several factors may contribute to this phenomenon. Firstly, since Hcy is linked to coronary restenosis [39] and slow blood flow [15], the potential benefits of Hcy-lowering therapies may have been obscured by the widespread use of antiplatelet agents and intensive lipid modulation, especially with the availability of newer-generation drug-eluting stents. Additionally, dietary variations across different regions lead to varying folate levels, which might influence the effectiveness of such therapies. Finally, the benefits of these therapies might only become apparent over longer treatment durations and extended follow-up periods. Folic acid and vitamin B are inexpensive and readily accessible, and their potential to lower Hcy levels and reduce cardiovascular risk warrants further exploration from a health economics standpoint. Given the low commercial incentives to test these affordable and non-patentable drugs, it is incumbent upon health authorities to take special responsibility in promoting and supporting such trials.

## Availability of Data and Materials

The datasets are available from the corresponding author on reasonable request.

## Supplementary Material

Supplementary material associated with this article can be found, in the online version, at https://doi.org/10.31083/RCM25518.
