# Supplementary material for: Prognostic Significance of Homocysteine Levels in Patients with ST-Segment Elevation Myocardial Infarction Undergoing Primary Percutaneous Coronary Intervention: A Propensity Score Matching and Weighting Analysis
Source: Rev Cardiovasc Med. 2025 Feb 19;26(2):25518. doi: 10.31083/RCM25518 (PMC11868880; doi:10.31083/RCM25518)
Supplement: Supplementary file 1 [file 2153-8174-26-2-25518-s1.docx]

**Supplementary Table 1** Results for Different PSM Models Adjusting for Various Covariates

| Adjustment Details | Number of Matched Participants | | HR | 95%CI | *p*-value |
| --- | --- | --- | --- | --- | --- |
|  | Normal Hcy | Elevated Hcy |  |  |  |
| Model 1 | 69 | 69 | 2.246 | 1.215-4.155 | 0.001 |
| Model 2 | 58 | 58 | 3.630 | 1.646-8.003 | 0.001 |
| Model 3 | 55 | 55 | 3.020 | 1.413-6.455 | 0.004 |
| Model 4 | 52 | 52 | 2.995 | 1.397-6.423 | 0.005 |

Model 1: Adjusted for age, sex, BMI, smoking, alcohol use, hypertension, diabetes, hyperlipidemia, and COPD.

Model 2: Adjusted as in Model 1 plus additional variables with a p-value < 0.05 in univariate Cox regression analysis (RBC, HB, HCT, D-dimer, serum creatinine, LVEF).

Model 3: Adjusted as in Model 1 plus variables with a p-value < 0.2 in univariate Cox regression analysis (HR, SBP, troponin, LDL-C, RBC, PLT, HB, HCT, D-dimer, serum creatinine, LVEF).

Model 4: Adjusted for all variables.

**Supplementary Table 2** Outcomes of patients stratified by Hcy

| Outcomes | Overall  (N = 183) | Normal Hcy  (N = 105) | Elevated Hcy  (N=78) | *p-*value |
| --- | --- | --- | --- | --- |
| MACE, n (%) | 55 (30.1%) | 19 (18.1%) | 36 (46.2%) | <0.001 |
| Cardiac death | 10 (5.5%) | 6 (5.7%) | 4 (5.1%) | 0.863 |
| Nonfatal myocardial infarction | 6 (3.3%) | 1 (1.0%) | 5 (6.4%) | 0.040 |
| Stroke | 4 (2.2%) | 1 (1.0%) | 3 (3.8%) | 0.186 |
| Ischemia-driven revascularization | 13 (7.1%) | 4 (3.8%) | 9 (11.5%) | 0.044 |
| Heart failure | 18 (9.8%) | 6 (5.7%) | 12 (15.4%) | 0.030 |
| All cause death | 4 (2.2%) | 1 (1.0%) | 3 (3.8%) | 0.186 |

Categorical variables were presented as number (percentage)

**Supplementary Table 3** Sensitivity Analysis Using E-value to Assess Robustness of Association Between Elevated Hcy Levels and MACE

| Analysis Method | HR | 95% CI | E-value for HR | E-value for CI |
| --- | --- | --- | --- | --- |
| Unadjusted | 2.778 | 1.591–4.850 | 5.000 | 2.561 |
| PSM | 2.995 | 1.397–6.423 | 5.439 | 2.142 |
| IPTW | 3.2 | 1.631–6.280 | 5.853 | 2.645 |

E-value represents the minimum strength of association that an unmeasured confounder would need to have with both the exposure (elevated Hcy levels) and the outcome (MACE) to fully explain away the observed association. The E-values for HR or CI were calculated using the following formula:

When HR > 1:

E-value for HR = HR + sqrt (HR × (HR - 1)).

For CI: if the lower limit (LL) ≤ 1, E-value = 1; if LL > 1, E-value = LL + sqrt (LL × (LL - 1)).

When HR < 1:

E-value for HR = (1 / HR) + sqrt [(1 / HR) × ((1 / HR) - 1)].

For CI: if the upper limit (UL) ≥ 1, E-value = 1; if UL < 1, E-value = (1 / UL) + sqrt [(1 / UL) × ((1 / UL) - 1)].


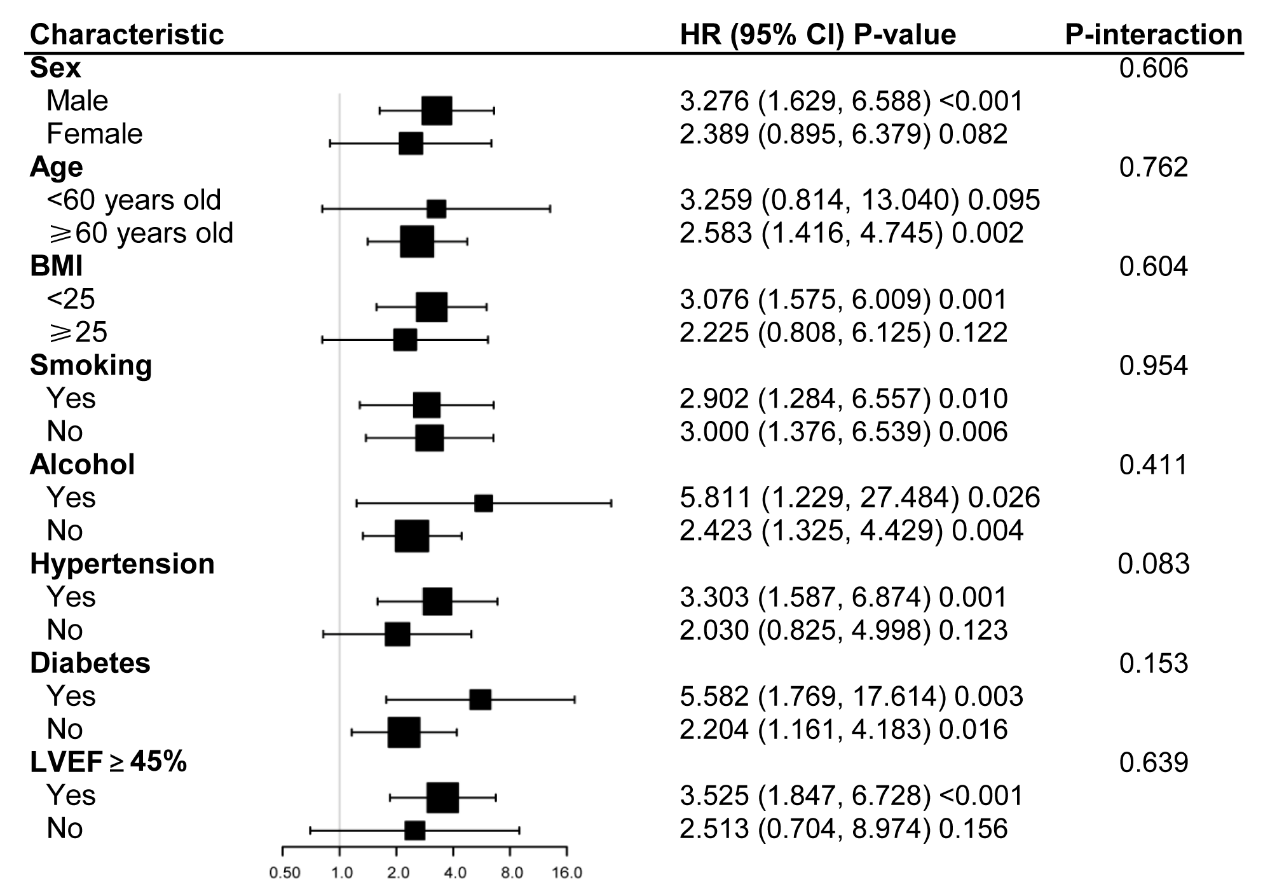


**Supplementary Fig. 1** Subgroup analysis of hazard ratios for MACE according to different baseline characteristics. The forest plot illustrates the HR and 95% CI for each subgroup analysis, including sex, age, BMI, smoking status, alcohol consumption, hypertension, diabetes, and LVEF.
